# Anti-protein arginine methyltransferase 5 (PRMT5) antibodies is associated with interstitial lung disease in rheumatoid arthritis

**DOI:** 10.1038/s41598-025-14741-2

**Published:** 2025-08-11

**Authors:** Renliang Huang, Jiaxi Guo, Qiaomiao Zhou, Heqing Huang, Shaowei Lin, Yikai Lin, Frank Petersen, Xinhua Yu, Aiping Ma

**Affiliations:** 1 Department of Genetics and Prenatal Diagnosis, Hainan Women and Children’s Medical Center, Haikou, Hainan China; 2 Department of Respiratory and Critical Medicine, School of Medicine, The First Affiliated Hospital of Xiamen University, Xiamen University, NO.55 ZhenHai Road, Xiamen, 361001 China; 3 Priority Area Chronic Lung Diseases, Research Center Borstel-Leibniz Lung Center, Member of the German Center for Lung Research (DZL), 23845 Borstel, Germany; 4 Department of Rheumatology, School of Medicine, The First Affiliated Hospital of Xiamen University, Xiamen University, Xiamen, China; 5 Department of Nuclear Medicine, School of Medicine, The First Affiliated Hospital of Xiamen University, Xiamen University, Xiamen, China; 6 Department of Radiology, School of Medicine, The First Affiliated Hospital of Xiamen University, Xiamen University, Xiamen, China

**Keywords:** Rheumatoid arthritis, Systemic sclerosis, Interstitial lung diseases, Anti-PRMT5 antibodies, Immunology, Rheumatology

## Abstract

Recently, anti-protein arginine methyltransferase 5 (PRMT5) antibodies has been identified a novel marker for systemic sclerosis (SSc). The present study aimed to to explore the association between anti-PRMT5 antibodies and rheumatoid arthritis (RA). The study recruited a cohort of 33 patients diagnosed with SSc, 87 patients with RA, and 31 healthy control subjects. Serum levels of the anti-PRMT5 antibodies were measured using a double-antigen sandwich enzyme-linked immunosorbent assay (ELISA). Patients with SSc exhibited higher serum levels (*p* < 0.001) and seropositivity rates (48.5% vs. 0%, *p* < 0.0001) of anti-PRMT5 antibodies compared to healthy subjects. Notably, serum levels of anti-PRMT5 antibodies were significantly elevated in patients with RA compared to healthy controls (*p* < 0.001), with a corresponding increase in the rate of seropositivity (14.9% vs. 0%, *p* < 0.05). Receiver operating curve analysis for anti-PRMT5 antibody levels yielded area under the curve values of 0.768 for distinguishing RA patients from healthy controls, and 0.903 for distinguishing SSc patients from controls. Furthermore, elevated levels of anti-PRMT5 antibodies were significantly associated with the presence of interstitial lung disease (ILD) (*p* < 0.0001) and antinuclear antibody positivity (*p* < 0.01). This study not only confirms the association of the anti-PRMT5 antibodies with SSc but also, for the first time, demonstrates a significant association between the anti-PRMT5 antibodies and RA, particularly in the context of RA-associated ILD.

**Supplementary Information:**

The online version contains supplementary material available at 10.1038/s41598-025-14741-2.

## Introduction

Connective tissue diseases (CTDs) such as rheumatoid arthritis (RA) and systemic sclerosis (SSc) are associated with autoantibodies, some of which serve as diagnostic biomarkers^1^. Very recently, anti-protein arginine methyltransferase 5 (PRMT5) antibodies have been identified as a novel biomarker for SSc^2^. The anti-PRMT5 antibodies are significantly elevated in patients with SSc and exhibits robust diagnostic accuracy in distinguishing SSc from healthy subjects^2^. Furthermore, the presence of anti-PRMT5 antibodies is associated with the progression of interstitial lung disease (ILD), particularly fibrosing ILD. In line with clinical observations, immunization with PRMT5 can induce significant inflammation and fibrosis in both the skin and lungs of mice, resembling key histological features of SSc^2^. Given these findings, the anti-PRMT5 antibodies have emerged as a promising autoimmune biomarker, meriting further investigation.

Interestingly, anti-PRMT5 antibodies have not been detected in two other CTDs, namely systemic lupus erythematosus (SLE) and Sjögren’s syndrome (SjS)^2^. RA, the most common autoimmune CTD, is also featured by pulmonary involvement, including ILD. Furthermore, several autoantibodies, such as those directed against Ro60 and Ro52, are commonly observed in both RA and SSc^1,3^. Therefore, it is plausible that the novel anti-PRMT5 antibodies are associated with RA and it pulmonary manifestation. In this study, we aimed to investigate the association between anti-PRMT5 antibodies and RA.

## Patients and methods

### Studied subjects

All study participants were recruited from the First Affiliated Hospital of Xiamen, Xiamen, China. Patients diagnosed with SSc or RA were hospitalized at the time of enrollment at the Department Rheumatology, while healthy controls were enrolled form Physical Examination Center. The diagnosis of RA adhered to the 2010 RA classification criteria proposed by the American College of Rheumatology (ACR)/European League Against Rheumatism (EULAR)^4^, while the diagnosis of SS was based on the 2013 ACR/EULAR criteria^5^. The exclusion criteria for SSc and RA groups included: (1) coexisting CTDs; (2) comorbid chronic pulmonary disorders; (3) malignancy; and (4) acute infections. Demographic and clinical characteristics, including age, sex, body mass index (BMI), disease duration and subtypes of SSc were retrieved from medical records. Autoantibodies, including anti-nuclear antibodies (ANA), rheumatoid factor (RF), anti-cyclic citrullinated peptide (anti-CCP), anti-topoisomerase antibodies (ATA), anti-centromere antibodies (ACA), anti-Ro60 and anti-Ro52, were determined at the time of diagnosis using methods summarized in Supplementary Tables 1 and were also recorded. This study was conducted in accordance with the 1964 Declaration of Helsinki and its subsequent amendments or similar ethical standards and was approved by the Ethics Committee of the First Affiliated Hospital of Xiamen University (No. 2024063 and No.2023031). Written inform consent was obtained from all participants in the study.

### HRCT evaluation

High-resolution computed tomography (HRCT) of the chest was reviewed and analyzed independently by two observers who were blinded to the clinical data to assess the ILD grade in patients with RA^6^. ILD was categorized into four grades: 0 = no ILD, 1 = indeterminate ILD (focal or unilateral ground-glass attenuation, focal or unilateral reticulation, or patchy ground-glass abnormality involving < 5% of the lung), 2 = mild ILD (changes affecting > 5% of any lobar region with non-dependent ground-glass or reticular abnormalities, diffuse centrilobular nodularity, non-emphysematous cysts, honeycombing, or traction bronchiectasis), and 3 = advanced ILD (bilateral fibrosis in multiple lobes associated with honeycombing and traction bronchiectasis in a subpleural distribution).

### Determination of anti-PRMT5 antibodies

The anti-PRMT5 antibodies were determined using a double-antigen sandwich enzyme-linked immunosorbent assay (ELISA) with the PRMT5 Ab ELISA Kit (Jiangsu Meimian Industrial Co., Ltd, China). The assay has a reported detection limit of 0.3 ng/ml for anti-PRMT5 IgG, with a sensitivity exceeding 95% and a specificity of 99.1%. The PRMT5 antigen used in the kit is a recombinant full-length protein expressed in insect cells, with a purity of > 90%. The assay was performed according to the manufacturer’s instruction. Briefly, a 96-well plate pre-coated with full-length PRMT5 was incubated with 1:20 diluted human serum samples at 37 °C for 30 min. Following this incubation, the plate was washed and further incubated with HRP-conjugated PRMT5 at 37 °C for 30 min. After washing, TMB substrate was added to each well, and the reaction was stopped by a stopping solution. Finally, the optical density (OD) values at 450 nm were measured using a microplate reader. A mouse anti-human PRMT5 IgG antibody was included in the kit as a reference of positive control (Supplementary Fig. 1). To minimize potential bias in autoantibody detection, all ELISA procedures were performed and evaluated by RH, who was blinded to the clinical data and group assignments of both patients and controls.

### Statistical analyses

The sample size was calculated using G*Power software, employing a two-tailed Fisher’s exact test to compare proportions of seropositivity between patient and control groups^7^. All other statistical analyses were performed using GraphPad Prism Software (version 10.0.2). The Kolmogorov–Smirnov test was utilized to assess the normal distribution of variables. The Student’s t-test was employed to determine statistical significance for normally distributed quantitative data; otherwise, the Mann–Whitney U test was applied. One-way analysis of variance (ANOVA) was used for comparisons involving more than two group means. Spearman correlation analysis was conducted to investigate the relationship between quantitative variables. For qualitative variables, statistical significance was determined using the chi-squared test when all expected values of the contingency table were greater than 5; otherwise, Fisher’s exact test was conducted. A *p*-value less than 0.05 was considered statistically significant.

## Results

### Demographic and clinical characteristics of patients and controls

A total of 33 patients with SSc, 87 patients with RA, and 31 healthy control subjects were recruited for this study. Detailed demographic and clinical characteristics of the study subjects are summarized in Table 1. The male-to-female ratio was comparable across groups (Control: 11/20; SSc: 13/20; RA: 27/60). The mean age was similar between the control and RA groups (60.3 ± 8.9 and 61.6 ± 10.8 years, respectively), whereas SSc patients were slightly younger (54.7 ± 13.5 years). The median disease duration was 2.0 years (interquartile range [IQR]: 1.0–8.5) in the SSc group and 8.0 years (IQR: 3.0–20.0) in the RA group.

**Table 1:** Demographical and clinical characteristic of subjects in this study.

	Control (*n* = 31)	SSc (*n* = 33)	RA (*n* = 87)
Male/female	11/20	13/20	27/60
Age in years (mean ± SD)	60.3 ± 8.9	54.7 ± 13.5	61.6 ± 10.8
Disease duration in years, median (Q1-Q3)	–	2.0 (1.0-8.5)	8.0 (3.0–20.0)
Autoantibodies			
ANA	–	90.9% (30/33)	31.0% (26/84)
ANA titer*, median (range)	–	1:640 (1:160–1:10000)	1:90 (1:80–1:1600)
RF	–	–	81.9% (68/83)
ACPA	–	–	86.3% (69/80)
ATA	–	51.5% (17/33)	–
ACA	–	9.1% (3/33)	–
Anti-Ro60	–	–	9.2% (8/87)
Anti-Ro52	–	–	10.4%(9/85)
RA-ILD grade			
0	–	–	29.9% (26/87)
1	–	–	25.3% (22/87)
2	–	–	26.4% (23/87)
3	–	–	18.4% (16/87)
SSc subtype			
lcSSc	–	66.7% (22/33)	–
dcSSc	–	33.3% (11/33)	–

*ANA was determined using an indirect immunofluorescence assay with HEp-2 cells, and a titer over 1:80 was considered positive. Descriptive statistics of ANA titer was performed with 22 SSc patients and 24 RA patients with recorded ANA titer values. RA, rheumatoid arthritis; SSc, systemic sclerosis; ILD, interstitial lung disease; ANA, antinuclear antibodies; RF, rheumatoid factor; ACPA, anti-citrullinated protein antibodies; ATA, anti-topoisomerase antibodies. ACA, anti- centromere protein B antibodies.

Among SSc patients, 66.7% had limited cutaneous systemic sclerosis (lcSSc), while 33.3% had diffuse cutaneous systemic sclerosis (dcSSc). ANA were detected in 90.9% of SSc patients, with 51.5% testing positive for anti-topoisomerase antibodies ATA and 9.1% for ACA. In RA patients, the seropositivity rates were as follows: ANA, 31.0%; RF, 81.9%; anti-CCP, 86.3%; anti-Ro60, 9.2%; and anti-Ro52, 10.4%. Severity of ILD in RA patients was graded from 0 to 3, with 29.9% showing no ILD and 25.3%, 26.4%, and 18.4% classified as grades 1, 2, and 3, respectively.

### Levels and seropositivity of anti-PRMT5 antibodies in patients and controls

**Fig. 1:**
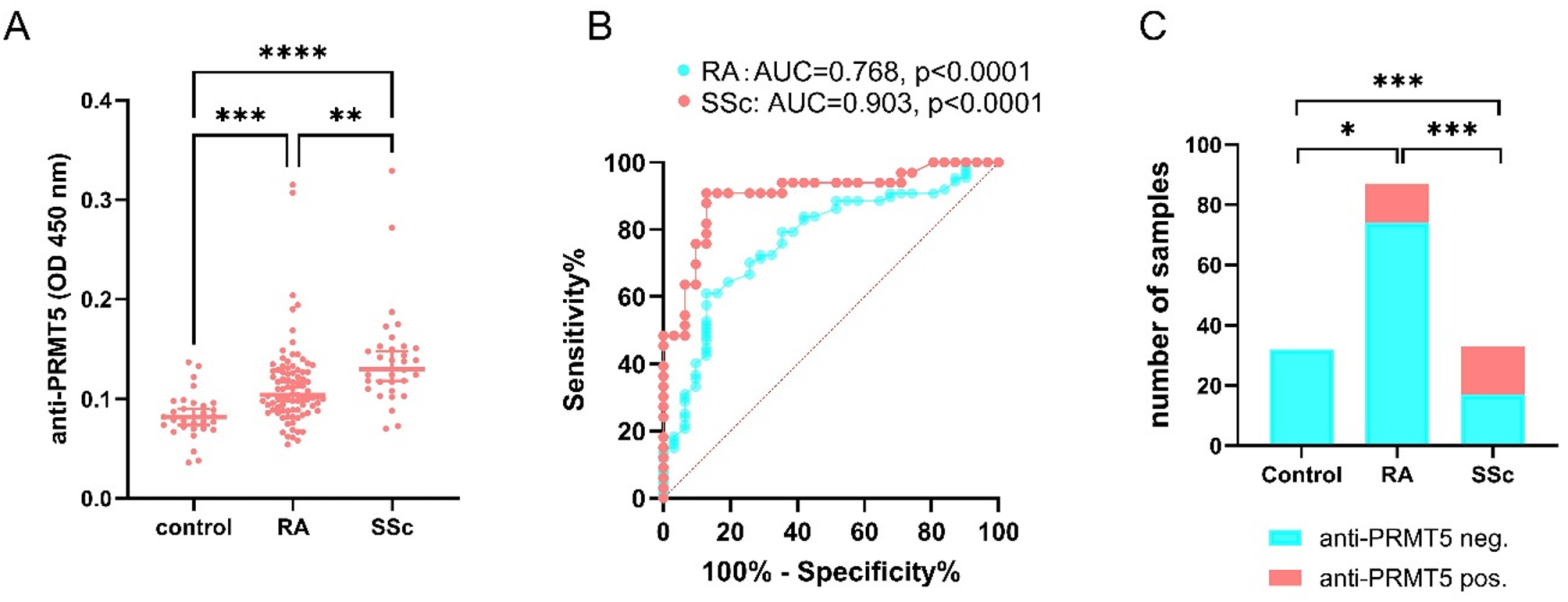
Association of anti-PRMT5 antibodies with systemic sclerosis (SSc) and rheumatoid arthritis (RA). Serum levels of the anti-PRMT5 antibodies were determined using a double-antigen sandwich ELISA. Seropositivity for the anti-PRMT5 antibodies was defined as OD 450 nm values above the 99th percentile of the upper limit observed in healthy controls (HC). (**A**) Serum levels of anti-PRMT5 antibodies in healthy controls (*n* = 31), patients with RA (*n* = 87) and patients with SSc (*n* = 33). (**B**) Receiver operating characteristic (ROC) curve analyses assessing the diagnostic performance of anti-PRMT5 antibodies in distinguishing RA and SSc patients from healthy controls. The area under the curve (AUC) and corresponding *p*-values are indicated. (**C**) Proportion of individuals seropositive for anti-PRMT5 antibodies in healthy controls, RA, and SSc cohorts. Statistical significance: *p < 0.05, **p < 0.01. *p* < 0.001 and ****p < 0.0001.

Serum levels of the anti-PRMT5 antibodies were determined using a double-antigen sandwich ELISA, and optical density at 450 nm (OD 450 nm) values were recorded for analysis. The median OD 450 nm value in healthy control subjects was 0.082, (range 0.036–0.137). In SSc patients, OD 450 nm values ranged from 0.07 to 0.329, with a median of 0.130, significantly higher than controls (*p* < 0.0001, Fig. 1A). Although lower than in SSc, anti-PRMT5 antibodies levels in patients with RA (median = 0.104, range 0.054–0.261) were also significantly elevated compared to healthy controls (*p* < 0.001, Fig. 1A). Receiver operating characteristic (ROC) curve analysis yielded area under the curve (AUC) values of 0.768 for RA (*p* < 0.0001) and 0.903 for SSc (*p* < 0.0001), indicating moderate to high discriminatory ability of anti-PRMT5 antibody levels in differentiating these diseases from controls (Fig. 1B).

When anti-PRMT5 antibody seropositivity was defined as OD 450 nm values above the 99th percentile of the healthy controls, none of healthy control subject (%) and 48.5% of SSc patients exhibited seropositivity (*p* < 0.0001, Fig. 1C). In RA patients, the rate of seropositivity for was 14.9%, significantly higher than that in healthy controls (*p* < 0.05), but lower than that in SSc patients (*p* < 0.001, Fig. 1C). Notably, among the 13 SSc patients who were double negative for ATA and ACA, 6 tested positive for anti-PRMT5 antibodies, suggesting potential additional diagnostic value of anti-PRMT5 in SSc (Supplementary Table 2).

Therefore, these findings not only confirm the strong association between anti-PRMT5 antibodies and SSc but also suggest a previously unrecognized association with RA.

### Association between anti-PRMT5 antibodies and clinical features of RA

**Fig. 2:**
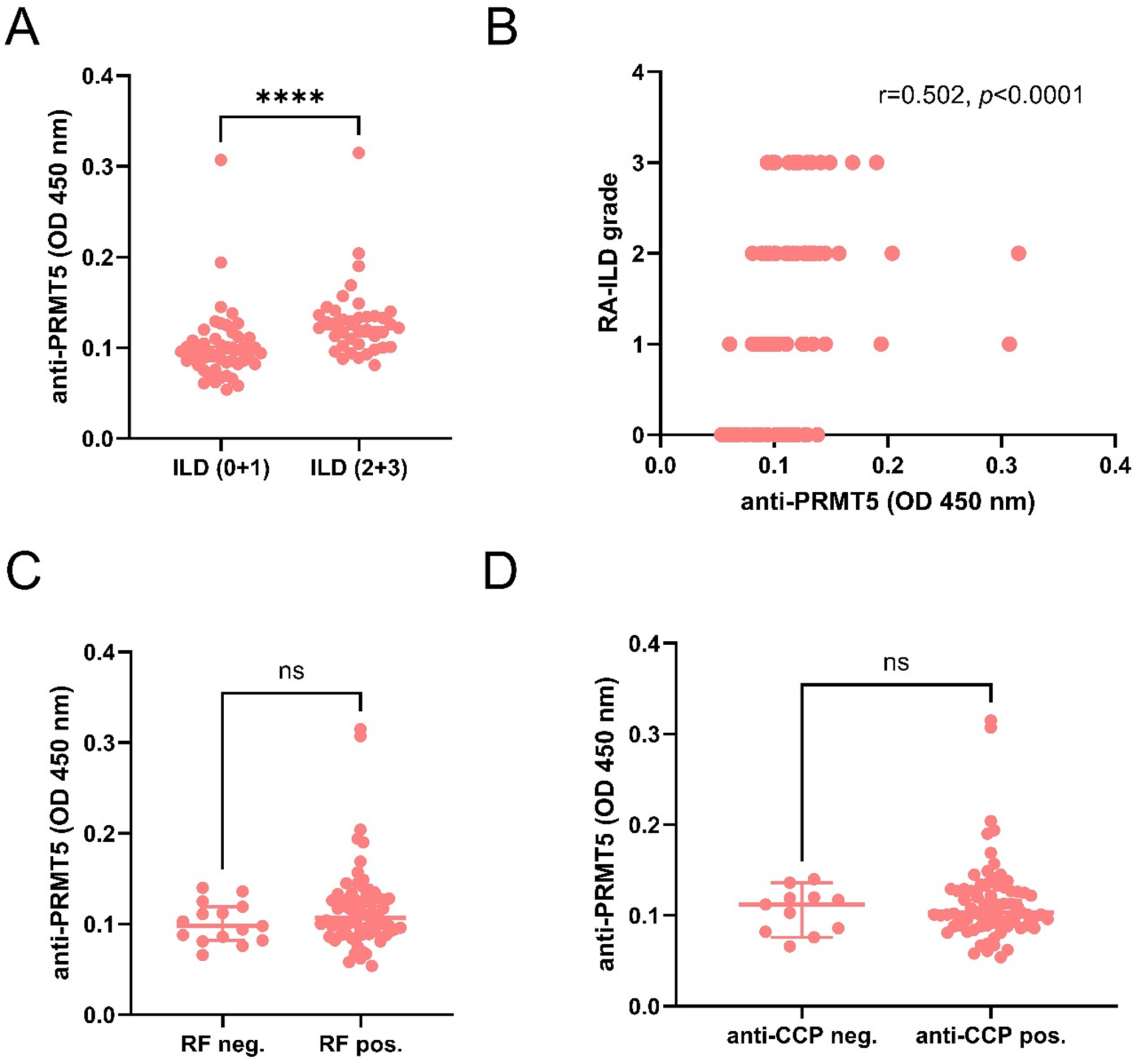
Association of anti-PRMT5 antibodies with clinical characteristics of RA. (**A**) Comparisons of serum levels of anti-PRMT5 antibodies between RA patients with no ILD or indeterminate ILD (RA-ILD: 0 + 1, *n* = 48) and RA patients with moderate or advanced ILD (RA-ILD: 2 + 3, *n* = 39). (**B**) Spearman correlation between anti-PRMT5 antibodies and ILD grades. Correlation coefficient (r) and *p*-value are indicated. Comparisons of serum levels of anti-PRMT5 antibodies between RF-negative RA and RF-positive RA patients (**C**) and between anti-CCP-negative and anti-CCP-positive RA patients (**D**). ns, not significant, ****p < 0.0001.

To further characterize the association between anti-PRMT5 antibodies and RA, we examined their relationship with clinical features of the disease. RA patients were categorized into two subgroups according to the severity of ILD: RA patients with no or indeterminate ILD and those with moderate to advanced ILD. Patients in the latter group showed significantly higher serum levels of the anti-PRMT5 antibodies compared to those in the former group (*p* < 0.0001, Fig. 2A). Consistent with this observation, a significant positive correlation was found between serum levels of anti-PRMT5 antibodies and the severity of ILD, as indicated by ILD grading (*r* = 0.502, *p* < 0.0001; Fig. 2B). In contrast, no significant associations were observed between anti-PRMT5 antibody levels and the two RA-associated diagnostic autoantibodies, RF and anti-CCP antibodies (Fig. 2C,D).

**Fig. 3:**
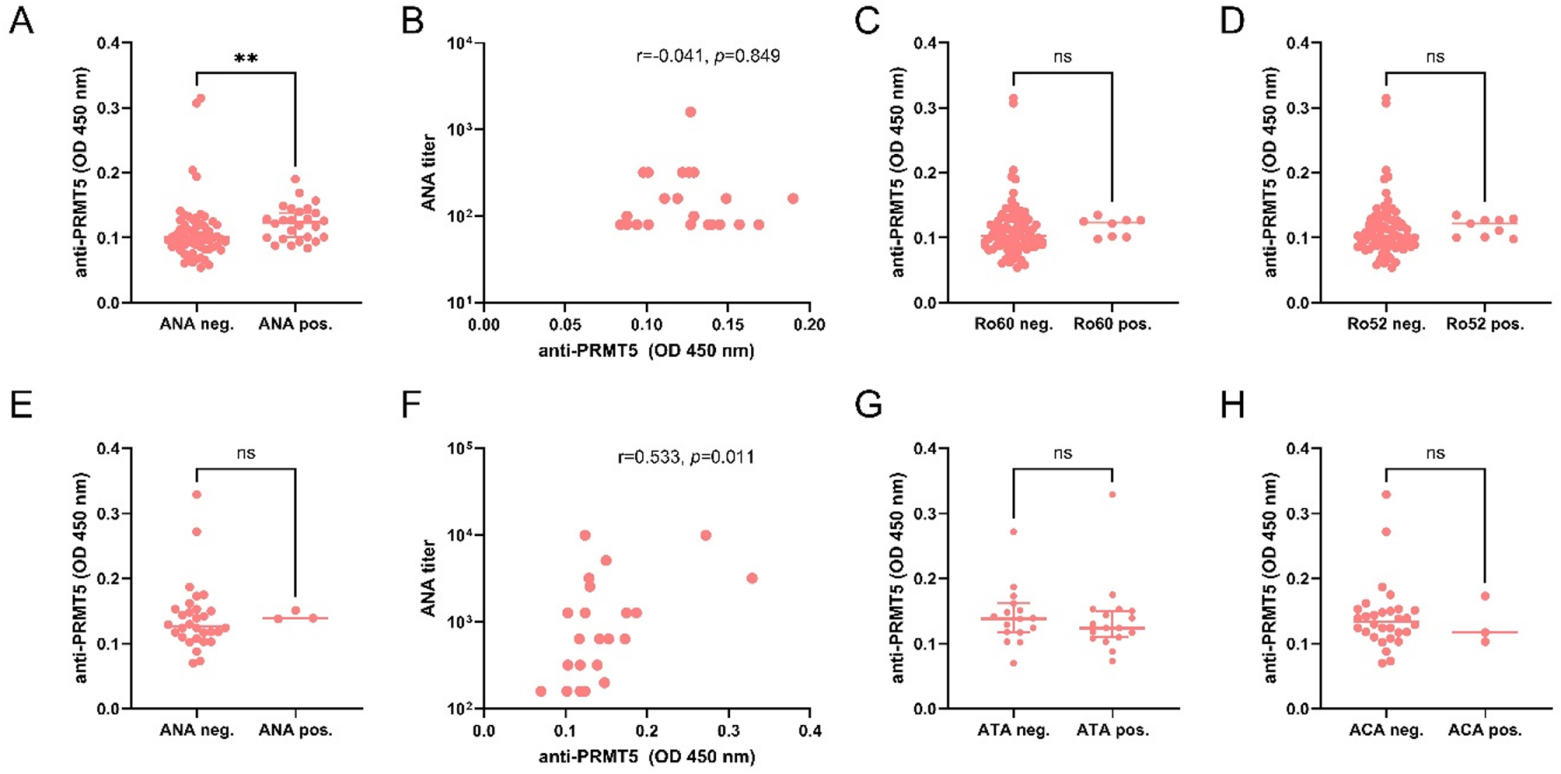
Relationship between anti-PRMT5 antibodies and antinuclear antibodies (ANA) in RA and SSc patients. (**A**,**E**) Comparison of serum anti-PRMT5 antibody levels between ANA-negative and ANA-positive patients in RA (**A**) and SSc (**E**). (**B**,**F**) Spearman correlation between anti-PRMT5 antibody levels and ANA titers in ANA-positive patients with RA (**B**) and SSc (**F**); correlation coefficients (r) and *p*-values are indicated. (**C**,**D**) Comparison of anti-PRMT5 antibody levels between Ro60-negative and Ro60-positive RA patients (**C**), and between Ro52-negative and Ro52-positive RA patients (**D**). (**G**,**H**) Comparison of anti-PRMT5 antibody levels between ATA-negative and ATA-positive SSc patients (**G**), and between ACA-negative and ACA-positive SSc patients (**H**). ns, not significant; **p < 0.01.

Notably, anti-PRMT5 antibody levels were significantly higher in ANA-positive RA patients compared to ANA-negative individuals (*p* < 0.01; Fig. 3A). However, no significant correlation was detected between anti-PRMT5 antibody levels and ANA titers in the RA cohort (Fig. 3B). Moreover, anti-PRMT5 levels were not significantly associated with the presence of Ro60 or Ro52 (TRIM21) autoantibodies (Fig. 3C,D).

In patients with SSc, no significant difference in anti-PRMT5 antibody levels was observed between ANA-positive and ANA-negative individuals (Fig. 3E). Nonetheless, a significant positive correlation was identified between anti-PRMT5 antibody levels and ANA titers (*r* = 0.533, *p* = 0.011; Fig. 3F). Additionally, anti-PRMT5 antibody levels showed no significant association with the presence of ATA or ACA in SSc patients (Fig. 3G,H).

## Discussion

In this study, we determined association of anti-PRMT5 antibodies with two connective tissue diseases, SSc and RA. Our findings reveal that serum levels of the anti-PRMT5 antibodies and the rate of seropositivity were significantly elevated in patients with SSc compared to healthy subjects. Additionally, we found no significant association between the anti-PRMT5 antibodies and specific subtypes of SSc or the presence of ATA. These observations are consistent with those reported by Liang et al.^2^. Unlike the classical sandwich ELISA utilized in Liang’s study, our research employed a double-antigen sandwich ELISA to detect the anti-PRMT5 antibodies. The double-antigen sandwich ELISA is a well-established method widely used for the detection of antibodies against various pathogens^8,9^. It needs to be mentioned that this method detects not only IgG antibodies but also other immunoglobulin classes. Therefore, our study validates the association of the anti-PRMT5 antibodies with SSc using a different, potentially more comprehensive, autoantibody detection approach.

Importantly, our study also establishes a novel association between the anti-PRMT5 antibodies and RA, as both antibody levels and seropositivity were significantly higher in RA patients compared to healthy controls. ROC curve analysis yielded high AUC values for both RA and SSc, suggesting that good predictive values of anti-PRMT5 antibodies in these diseases. However, the absolute OD 450 nm values observed in patient samples were relatively low. Given that high OD 450 nm values were achieved with higher concentrations of the positive control, it is unlikely that the low values in patient samples were due to technical limitations of the assay. Therefore, the relatively low OD 450 nm values may reflect either (1) low circulating levels of anti-PRMT5 antibodies or (2) low binding affinity of these antibodies in patient sera.

Stratified analysis further revealed that RA patients with ILD exhibited higher levels of the anti-PRMT5 antibodies than those without ILD, suggesting a potential role of this antibodies in the pathogenesis of RA-ILD. Accumulating evidence indicates that RA-associated autoantibodies, including RF and ACPA, are linked to the development of RA-ILD^10,11^. Recently, Kronzer et al. identified six fine-specificity antibodies against citrullinated or native proteins as being associated with RA-ILD^12^. The association between the anti-PRMT5 antibodies and RA-ILD identified in our study provides additional support for the role of autoantibodies in RA-associated lung disease.

The presence of anti-PRMT5 antibodies in both SSc and RA, and their associated with the disease, is notable and suggests that the autoantibodies may also be present in other CTDs. Given that anti-PRMT5 antibodies were associated with presence of ILD in RA, their presence and clinical significance in other ILD-associated CTDs is of particular interest. In addition to SSc and RA, several other CTDs are frequently associated ILD, including polymyositis/dermatomyositis, mixed CTD, SLE and SjS^13^. Although Liang et al. reported the absence of the anti-PRMT5 antibodies in SLE or SjS^2^, the observation was based on a a relatively small sample size (30 SLE and 9 SjS patients). Therefore, larger studies are warranted to clarify the potential association of anti-PRMT5 antibodies with these and other CTDs, particularly those complicated by ILD.

PRMT5, a well-known epigenetic regulatory enzyme, is responsible for the methylation of various proteins, including histones, transcription factors, and cell receptors, thereby playing a crucial role in a wide range of biological processes^14^. Beyond its physiological functions, PRMT5 has been implicated in various pathological conditions, such as cancer and inflammatory diseases^14^. It has been shown that PRMT5 is overexpressed in B cells and is essential for their development, activation, and proliferation^15^, underscoring its importance in B cell biology. Additionally, fibroblast-specific deficiency of PRMT5 has been found to suppress fibrosis by regulating TGF-β/Smad3-dependent fibrotic gene transcription^16^, indicating a key role for PRMT5 in tissue fibrosis. Given that immunization with PRMT5 can induce pulmonary fibrosis in mice^2^, it is plausible that immune responses against PRMT5 contribute to the fibrotic process in the lungs.

This study has several limitations. First, unlike classical ELISA formats, the double-antigen sandwich ELISA used in this study does not allow for the determination of antibody isotypes. As a result, the detected anti-PRMT5 antibodies represent a mixture of immunoglobulin classes (e.g., IgG, IgM, and IgA), limiting our ability to characterize the specific immune response. Second, due to the absence of established confirmatory assays such as immunoprecipitation or immunoblotting, the anti-PRMT5 positivity observed in this study could not be independently validated, which may affect the robustness of our findings. Third, the relatively small sample size and the single-center design may limit the generalizability of the results. Further multi-center studies with larger cohorts and methodological validation are warranted to confirm and extend these findings.

In conclusion, this study confirms the presence of the anti-PRMT5 antibodies and their association with SSc in an independent cohort. Furthermore, our findings demonstrate that this anti-PRMT5 antibodies are also associated with RA and correlates with the presence of ILD and ANA in these patients.

## Supplementary Information

Below is the link to the electronic supplementary material.

Supplementary Material 1

## Data Availability

Data are available from the correspondence author upon reasonable request.
