# Supplementary material for: Anti-protein arginine methyltransferase 5 (PRMT5) antibodies is associated with interstitial lung disease in rheumatoid arthritis
Source: Sci Rep. 2025 Aug 11;15:29421. doi: 10.1038/s41598-025-14741-2 (PMC12340103; doi:10.1038/s41598-025-14741-2)
Supplement: Supplementary file 1 — Supplementary Material 1 [file 41598_2025_14741_MOESM1_ESM.docx]

**Supplementary information**

**Supplementary Table 1. Summary of methodologies used for the detection of autoantibodies.**

| **Antibody** | **Method** | **Kit** | **Manufacture** |
| --- | --- | --- | --- |
| ANA | Indirect immunofluorescence | ANA (HEp-2) Test System | Aesku, Germany |
| RF | Turbidimetric immunoassay | N Latex RF Kit | Siemens Healthineers, Germany |
| Anti-CCP | ELISA | Anti-CCP ELISA | Euroimmun, Germany |
| ATA | Immunoblotting | EUROLINE ANA Profile | Euroimmun, Germany |
| ACA | Immunoblotting | EUROLINE ANA Profile | Euroimmun, Germany |
| Ro60 | Immunoblotting | EUROLINE ANA Profile | Euroimmun, Germany |
| Ro52 | Immunoblotting | EUROLINE ANA Profile | Euroimmun, Germany |

RA. rheumatoid arthritis; SSc. systemic sclerosis; ANA. antinuclear antibodies; RF. rheumatoid factor; anti-CCP. anti-cyclic citrullinated peptite; ATA. anti-topoisomerase antibodies. ACA, anti- centromere protein B antibodies.

**Supplementary Table 2. Association between anti-PRMT5 antibodies and other diagnostic autoantibodies in patients with systemic sclerosis.**

|  |  | Anti-PRMT5 negative | Anti-PRMT5 positive |
| --- | --- | --- | --- |
| SSc | All (n=33) | 20 | 13 |
|  | ATA-ACA- (n=13) | 7 | 6 |
|  | ATA+ACA- (n=17) | 11 | 6 |
|  | ATA-ACA+ (n=3) | 2 | 1 |
|  | ATA+ACA+ (n=0) | 0 | 0 |

SSc. systemic sclerosis; ATA. anti-topoisomerase antibodies; ACA, anti- centromere protein B antibodies; PRMT5. protein arginine methyltransferase 5.


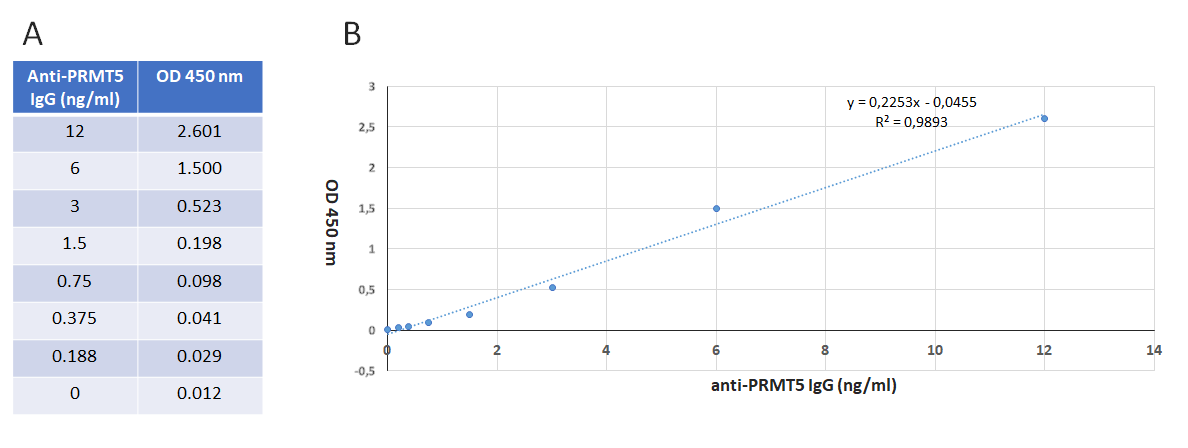


**Supplementary Figure 1. Performance of anti-PRMT5 IgG as a reference standard in ELISA.** A. Optical density (OD) values at 450 nm obtained from ELISA using serial dilutions of anti-PRMT5 IgG. B. Standard curve generated from the ELISA OD450 readings of anti-PRMT5 IgG, demonstrating the assay's dynamic range and linearity.
